# Association between the ambient temperature and the occurrence of human *Salmonella* and *Campylobacter* infections

**DOI:** 10.1038/srep28442

**Published:** 2016-06-21

**Authors:** Josef Yun, Matthias Greiner, Christiane Höller, Ute Messelhäusser, Albert Rampp, Günter Klein

**Affiliations:** 1 Institute of Food Quality and Food Safety, University of Veterinary Medicine of Hannover Foundation, Bischofsholer Damm 15, D-30173 Hannover, Germany; 2 Government of Lower Bavaria, Regierungsplatz 540, D-84028 Landshut, Germany; 3 Federal Institute for Risk Assessment (BfR) and University of Veterinary Medicine of Hannover Foundation, Max-Dohrn-Str 8-10, D-10589 Berlin, Germany; 4 Bavarian Health and Food Safety Authority, Veterinärstr. 2, D-85764 Oberschleissheim, Germany

## Abstract

*Salmonella* spp. and thermotolerant *Campylobacter* spp. are the most important causes of human bacterial diarrheal infections worldwide. These bacterial species are influenced by several factors like behaviour of the host, shedding, environment incl. directly or indirectly through ambient temperature, and the infections show seasonality. Therefore, the aim of our study was to investigate the association between the occurrence of human campylobacteriosis and salmonellosis and the ambient temperature. The number of campylobacteriosis and salmonellosis cases in two German metropolises, Munich and Berlin, and three rural regions was analysed with simultaneous consideration of the ambient temperature over a period of four years (2001 to 2004) using regression, time series, and cross-correlation analysis. The statistical analysis showed that an increase in the ambient temperature correlated positively with an increase in human *Salmonella* and *Campylobacter* cases. The correlation occurred with a delay of approximately five weeks. The seasonal rise in ambient temperature correlated with increased incidence of bacterial diarrheal infections.

*Salmonella* spp. and thermophilic *Campylobacter* spp. are the most important causes of human bacterial diarrheal infections in Europe and worldwide. In 2013, the European Food Safety Authority (EFSA) and the European Centre for Disease Prevention and Control (ECDC) reported approximately 215.000 cases of human campylobacteriosis and 85.000 cases of human salmonellosis in Europe^1^. At the species level, *Campylobacter* (*C.*) *jejuni* subsp. *jejuni,* followed by *C. coli,* are most commonly associated with human diseases. The *Salmonella* serotypes, *Salmonella* (*S.*) serotype Enteritidis and *S.* ser. Typhimurium, are the most frequently isolated in cases of human salmonellosis. Seasonality as a defining impact on incidence has been considered for human salmonellosis^2^,^3^,^4^,^5^ and campylobacteriosis^6^,^7^,^8^,^9^,^10^ as well as for other foodborne pathogens^11^,^12^,^13^,^14^, emphasizing the public health importance of seasonal outbreak patterns. However, the studies do not show unambiguously seasonality, as geographical, climatic or production related factors can lead to different results. The seasonality of campylobacteriosis, if present, correlates to seasonality of *Campylobacter* colonisation in commercial broiler flocks^15^. For *Salmonella* the correlation between the occurrence of salmonellosis in humans and seasons is often explained by barbecuing or gardening, not by the contamination of chickens^16^, but also shedding by other farm animal species like pigs and cattle must be taken into account. Except of one study on *Campylobacter* time trend in broiler and humans^9^ the association between the occurrence of human cases and temperature has not been studied in Germany.

The hypothesis of our study was that there is an association between the occurrence of human campylobacteriosis and salmonellosis and the ambient temperature. We therefore investigated data in municipal and rural German study sites. Specifically, we sought to identify the lag time, in weeks, for which the association between incidence and temperature was maximal. We therefore think that our study contributes to a better understanding of the association between the occurrence of clinical cases and the ambient temperature.

## Materials and Methods

The study was performed according to German law and has been approved by the competent committee of the University of Veterinary Medicine, Hannover.

### Prevalence of human campylobacteriosis and salmonellosis

Information on the weekly frequency of human campylobacteriosis and salmonellosis cases was obtained from the reports of the Robert-Koch-Institut (RKI) from 2001 to 2004 for metropolitan (Berlin, Munich) and rural study sites located in North-Swabian counties (Dillingen, Donau-Ries und Augsburg-Land)^17^. Eligible cases reported by the RKI had to meet at least one of the following criteria according to the German Infection Control Law of 2001:

- clinical symptoms and laboratory confirmed
- clinical-epidemiologically confirmed
- clinically confirmed

The reporting period from the year 2001 onwards has been chosen, because a new reporting scheme was established in Germany in 2001 according to the Infection Control Law. This reporting scheme did not change through the whole reporting period, as well as no changes in the detection and reporting methods occurred. In the following, the incidences and case rates refer to the absolute counts of reported cases as described above.

### Climate information

Temperature measurements were obtained from Germany’s National Meteorological Service (DWD) for five locations: Berlin, Munich, Neuburg, Augsburg and Dillingen: from 2001 to 2004 (1461 days in total)^18^. DWD calculates the temperature average for every day from 24 temperature values, which are measured every hour. In situations where more than three temperature values were missed in a single day, the temperature average is calculated from the four values collected as 00, 06, 12, 18 Coordinated Universal Time (UTC). The reference time for one day starts normally at 23.51 UTC of the day before and ends at 23.50 UTC of the same day. Observation dates were related to the global used time in Greenwich (Greenwich Mean Time (GMT) or UTC). The fixed date of observation is always 10 minutes before the reference point and the measurement is performed two meters above ground^18^. The daily mean temperature data were aggregated to determine mean and maximum weekly temperatures, resulting in results for 212 weeks covering the study period. Geographical variability was expressed using the between-location standard deviation (SD) of mean temperatures for each of the 212 weeks. Likewise, between-location SD was established for 212 weekly maximum temperatures.

### Statistical methods

**Figure 1:**
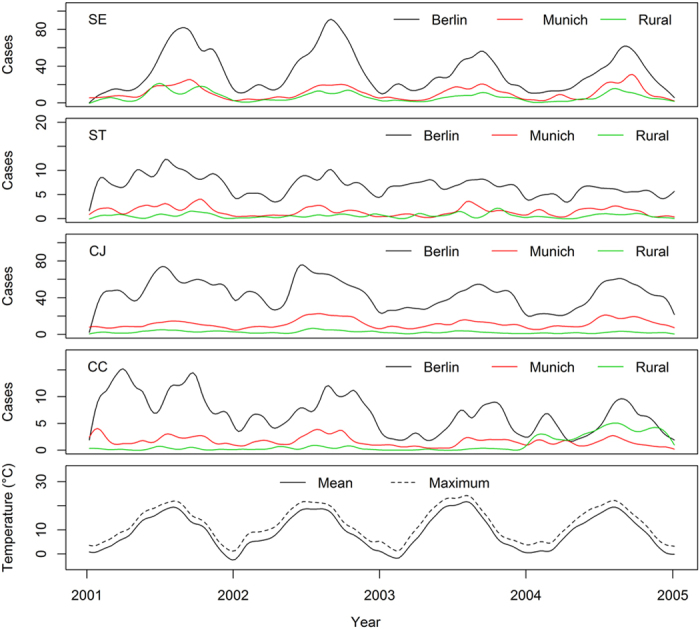
Smoothed time series for weekly incidences of human *S.* Enteritidis (SE), *S.* Typhimurium (ST), *Campylobacter* (*C.*) *jejuni* (CJ) and *C. coli* (CC) cases reported in three areas (data obtained from Robert-Koch Institut, RKI, Germany) and average weekly temperature (data obtained from Germany’s Meteorological Service, DWD) in Germany 2001–2004.

**Figure 2:**
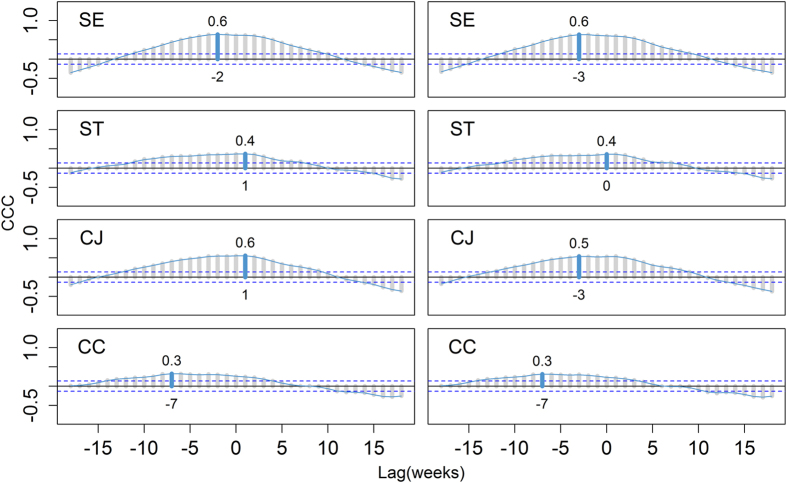
Cross-correlation coefficient (CCC) analysis on human diarrheal infection. Weekly reported incidences of *S.* Enteritidis (SE), *S.* Typhimurium (ST), *Campylobacter* (*C.*) *jejuni* (CJ) and *C. coli* (CC) in three reporting areas were correlated with mean (left column) and maximum (right column) weekly temperature recordings in Germany 2001–2004. A smoothed spline is fitted to the CCC values (grey lines). The lag time (week) at which maximum correlation is observed and the corresponding CCC are indicated (values below and above grey bars, negative lags correspond to disease peaks occurring after temperature peaks). The values outside the two horizontal (blue, dashed) lines indicate autocorrelations which are significantly different from zero.

The total reported cases per week of *S.* Enteritidis (SE), *S.* Typhimurium (ST), *Campylobacter* (*C.*) *jejuni* (CJ) and *C. coli* (CC), as well as the mean and maximum weekly temperature, were plotted as smoothed time series (Fig. 1) using the open source software package, R (command smoothed.spline with a smoothing parameter of 0.4 in the R statistical programme^19^). Lag time was investigated between recorded temperature (weekly mean and maximum values) and reported cases using cross correlation analysis (R command ccf) yielding a time-series cross-correlation coefficient (CCC) (Fig. 2). To remove any possible trend effect, this analysis uses detrended and z-transformed case data (see details in supporting information). Briefly, we fitted generalized linear models with log-link and negative binomial errors and a continuous time variable as predictor for the number of cases for each pathogen and study area and used the model residuals and observed mean number of cases for detrending the data. Lag time (in weeks) was identified for which a smoothed spline fitted to the observed CCC values became maximal.

**Figure 3:**
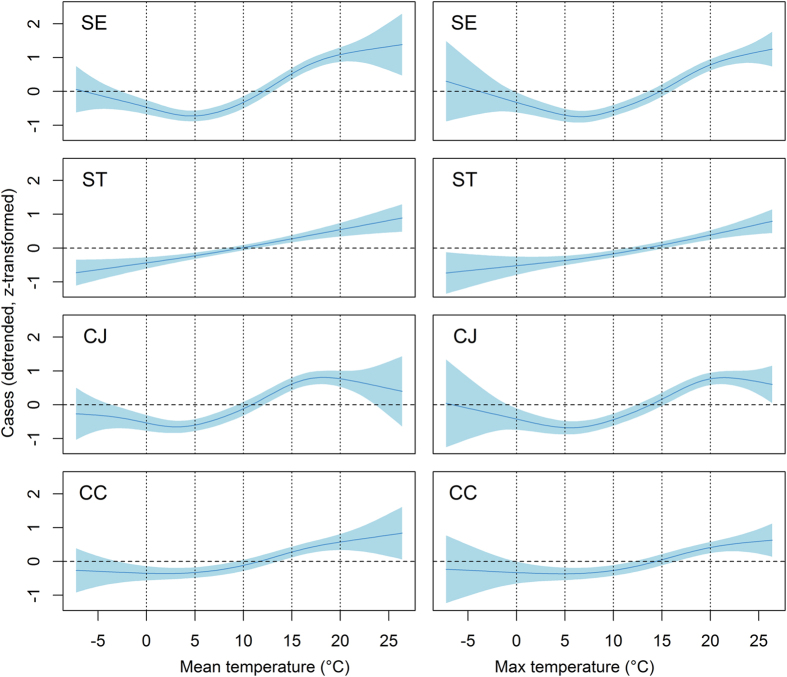
Smoothed regression splines and 95% uncertainty envelope of the weekly incidences of *S.* Enteritidis (SE), *S*. Typhimurium (ST), *Campylobacter* (*C.*) *jejuni* (CJ) and *C. coli* (CC) in three reporting areas and mean (left) and maximum (right) weekly temperature recording in Germany 2001–2004. Weekly incidences were z-transformed (mean subtracted and divided by standard deviation) and lagged by 5, 0, 4 and 7 weeks for incidences of SE, ST, CJ, and CC, respectively, to illustrate the maximum correlation (see Fig. 2).

Using the time lag in weeks that resulted in maximum correlation, the case rates were plotted against the mean and maximum temperature for the four-year lagged time series (Fig. 3). To account for trend effects and to achieve comparability among pathogens with different incidence levels, the case rates were detrended and z-transformed (detrended rate minus mean rate, and then divided by SD of the detrended rate). The trends and the uncertainty were visualised using a smoothed regression spline and 95% confidence envelope based on a generalised additive model (“gam” method from mgcv package^20^ in R (1209).

We built a negative binomial regression model using a training data set obtained by randomly splitting the data set into two equal parts. Using the training data, we tested the effect of temperature at various lag periods and moving average, resulting in the choice of a lag-optimised temperature model. This model uses the variable *top*, containing three-week moving average temperature at specific lag that provided the best according to Akaike’s information criterion (AIC) for each combination of location and bacterial species. We included into the linear predictor also the bacterial agent (SE as reference compared to ST, CJ and CC), the location (B as reference compared to M), the time in weeks as well as interaction terms between bacterial agent and all other predictors. An alternative model has a similar form but used a common-lag temperature that provided the best fit (AIC) for all combinations of location and bacterial species. Case data reported form the rural locations were omitted from these models because they were composed of three smaller (geographically distinct) recruiting areas with small numbers of cases. Since R-square cannot be computed for negative binomial regression models, we used McFadden’s pseudo R-square as a surrogate estimate of the proportion of variance explained by the model. See supporting material for details on the statistical analyses.

## Results and Discussion

First, we asked whether the temperature recordings across the geographic locations varied widely. If the temperature variation was minimal, this would allow us to simplify the preliminary analysis using a single set of temperature values for all study sites. We expressed the geographical variability of temperature using the between-location standard deviation (SD) of mean temperatures for each of the 212 weeks of the study. Likewise, between-location SD was established. The between-location SD for all 212 weekly mean and maximum temperatures had a median (and 95^th^ percentile) of 1.0 and 1.2 °C (2.1 and 2.4 °C), respectively, indicating only little temperature variation among the four locations. Therefore, we averaged the weekly mean and maximum temperatures values for the locations for the further analysis except for the final model.

To observe changes in the frequency of SE, ST, CJ and CC human cases reported in three study areas (Berlin, Munich and rural areas) with respect to time, we plotted the number of cases as a function of time. We found that incidence of all four types of infections peaked almost simultaneously, showing annual seasonality with peaks in summer, whereby the pattern of peaks was most pronounced for Berlin, less pronounced for Munich and weakest in rural areas (Fig. 1). A thorough analysis of the reporting practices could allow an adjustment for underreporting rates but this was outside the scope of this study. Since the location of peaks on the time scale was similar for the three locations, the case data from Berlin, Munich and rural areas were combined for further analyses. The seasonality was particularly strong for SE and CJ and moderate for ST and CC. These findings are in accordance with seasonality of incidences of *Salmonella* and *Campylobacter* reported elsewhere^1^,^2^,^3^,^4^,^5^,^6^,^7^,^8^,^9^,^10^. Various explanations have been given for this observation. First, *Salmonella* is able to multiply at ambient temperatures with generation times as low as 20 min. However the growth rate of *Salmonella* is increased at 30 °C and higher. Alternatively, in the summer months cross-contamination and/or undercooking of meat is more likely to occur, e.g. by barbecuing outdoors. *Campylobacter* is more prevalent from spring through autumn in food producing animals, but especially in poultry^1^,^7^. One hypothesis for this observation may be the contamination of flocks with flies, which can transmit *Campylobacter* spp.^7^ or increased agricultural activity^8^.

One of the Bradford-Hill criteria to demonstrate the assumed causality is to establish a time delay between the putative cause and the hypothetical effect. Therefore, we investigated the parallel time series for temperature (mean and maximum values) and incidence using cross correlation analysis. We sought to identify the lag time, in weeks, for which the correlation was maximal. Cross-correlation coefficients (CCC) for the investigated lag times showed a slight non-monotonic behaviour; the maximum was identified after smoothing the data. We found the lag times that resulted in maximum correlation (detrended and z-transformed case data and mean temperature) were 5, 3, 4, and 7 weeks for SE, ST, CJ and CC, respectively (later referred to as “maximum lags”) (Fig. 2). The CCC profiles for the three aetiologies SE, CJ and CC differed from the profile for ST in three important aspects. First, these CCC profiles appeared more peaked; second, higher correlation coefficients at the maximum lag were found except for CC and third, greater maximum lags were found. These results indicate that, for all aetiologies except ST, correlation between incidence of infection and increased temperature patterns aligned maximally few weeks before the increase in incidence. This provides empirical support for the hypothesis of an assumed causal correlation of the ambient temperature with the observed case rates for SE, CJ and CC infections. Such effect of temperature on ST cases is not supported by our data. The maximum observed CCC values were 0.8, 0.6, and 0.4 for SE, CJ, and CC, respectively, reflecting moderate correlation. The underlying mechanisms leading to the observed maximum lags are unknown. All of the aetiologies investigated here are known to cause food-borne infections in humans. Time differences ranging from 0 to 30 days between onset of clinical signs and date of reporting in various European regions have been reported^2^, suggesting a variation among different populations and surveillance systems. The size of the temperature effect, estimated by the same authors, exceeded a 10% increase in human cases per degree centigrade for some of the regions. A comparison with lag periods reported in other studies (e.g. refs 2, 6) is difficult due to potential differences in reporting.

**Figure 4:**
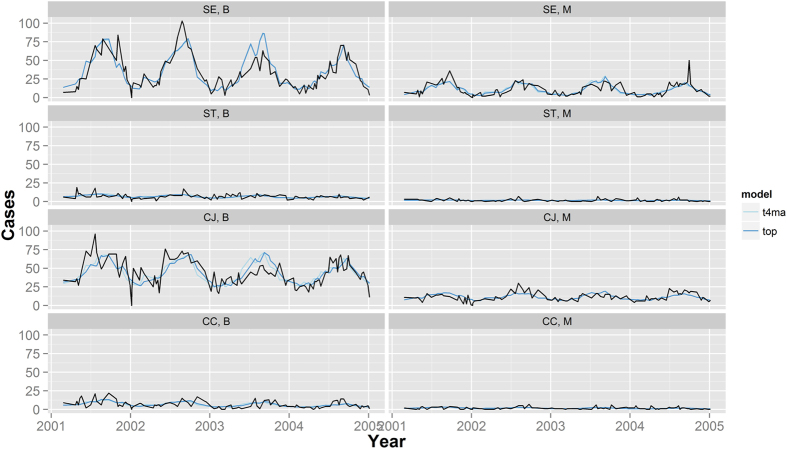
Weekly incidences of *S.* Enteritidis (SE), *S*. Typhimurium (ST), *Campylobacter* (*C.*) *jejuni* (CJ) and *C. coli* (CC) reported in Berlin (B) and Munich (M) 2001–2004 and overlaid predictions based on negative bionomial regression lag-optimised temperature model (using the variable *top*) and a common-lag temperature model (using the variable *t4ma*).

We investigated the pattern of correlation between ambient temperature and infection incidence. Correlation coefficients cannot capture non-linear correlation patterns. Therefore, we visualised the correlation pattern using a smoothed regression spline. Averaged data from our time series plots (Fig. 1, bottom panel) showed that the effect of temperature was similar over all four years of our study. Thus, it was possible to combine the weekly data from all four years for this analysis. The smoothed regression splines of the detrended and z-transformed case data revealed a non-linear relationship between temperature and infection incidence for all aetiologies, except for ST, which showed a more linear relationship. These results suggest that SE, CJ, and CC incidences are not affected at temperature below 5 °C. The incidences appear to increase steadily at temperatures higher than 5 °C. For CJ infection, there is some indication that incidences plateau at temperatures above about 18 °C. We found no clear difference between results obtained using mean and maximum weekly temperatures, suggesting that the mean temperature is a good predictor for the incidence rates. Finally, we demonstrated the combined effect of location-specific temperature adjusted for a potential trend using negative binomial regression. The lag-optimised temperature model (using variable *top*) accounted for different optimal lag periods for each combination of study location and bacterial species. The lags that provided the best fit (AIC) were 4, 5, 1, 2, 6, 4, 6 and 5 weeks for B/SE, M/SE, B/ST, M/ST, B/CJ, M/CJ, B/CC, M/CC, respectively. The best fit (AIC) with a common-lag temperature model was achieved with a three-week moving average temperature centred at 4-week (variable *t4ma*). The graphical results demonstrate the fit of the models (Fig. 4). The proportion of variance explained by the models (estimated using McFadden’s pseudo R-square) identified two time series, for which a linear trend over time explains more than 1% of variation (B/ST, B/CC) and two series for which linear trend and temperature explain more than 10% of variation (B/SE, M/SE) (Table 1). The percent variation explained by trend and temperature is between 2.7 and 9.7 for the remaining series. These results provide a crude approximation for – but are not identical with – the coefficient of determination available for ordinary least square regression models.

A long shelf-life period is often demanded from retailer and the consumer, thus reducing safety margins. In the case of *Salmonella* spp., inappropriate storage conditions that expose food stuff to increased ambient temperatures will lead to increased bacterial burden that can result in foodstuff that is not fit for human consumption. In the case of *Campylobacter* spp., the shelf life is less critical because bacterial multiplication is not possible in food alone according to present knowledge^7^. However, depending on the initial bacterial load and the storage temperature, the survival of *Campylobacter* spp. can be prolonged^21^,^22^. Additional risk factors for *Campylobacter* infections include surface water (recreational activities), consumption of raw milk, wild bird population, which can also be a reason for seasonality of human cases^7^. Nevertheless, food producers should consider the impact of seasonality on their products’ safety characteristics. In some cases unexpected seasonal patterns can occur. A study in Switzerland detected a winter peak in human campylobacteriosis cases, which was not related to temperature. The study identified a seasonal meat product (“Fondue chinoise”) as a major driver of the epidemic in the winter season^23^.

When using meteorological data for different regions two aspects are important. First the validity of the data must be considered and if they are derived from local weather stations as in our study or if they are gridded estimates of surface meteorological conditions^24^,^25^,^26^. The data from our study are very reliable as they are taken from local weather stations by the official German Weather Office^18^. The second aspect to consider is if to use daily or weekly data (as in our study) or aggregated monthly aggregated data^27^. More robust information can be obtained when using actual local data derived from shorter time intervals. The limitations are the reported human cases for a given region, as they are normally reported on a weekly basis^17^.

### Limitations of the study

The reporting of cases of human illness concerning campylobacteriosis is legally required in most countries incl. Germany. However, it is well known that not all cases are actually reported^1^. The major reasons for underreporting are that only a fraction of actual cases are presented to a physician, that stool samples are not collected for analysis in all cases and that that microbiological analysis may fail to detect the pathogen. Furthermore, travelling abroad or incoming travellers are not separately monitored and their attribution to the overall cases could not be estimated. The use of absolute case counts instead of proper incidence rates for the population at risk is a further limitation of this study. Reliable demographic data for the study locations and time period are required for a conversion from counts to incidence and – in the ideal case–travel-associated cases should be accounted for. It should also be considered that since the time of the study period (2001–2004, reasons for choosing this period see Materials and Methods) there occurred changes to the German population and food consumption patterns. Also the time span of the study period (4 years) may be a limitation. Our study data do not permit differentiating among different transmission routes and identification of cases as belonging to one outbreak, which limits the fraction of case that can be explained by a temperature effect^28^. Finally, it is noted that this study investigates the association between annual patterns of temperature and incidences of four foodborne infections. Although the time delay between high temperature and incidence satisfies one of the Bradford-Hill criteria for causality, our results suggest that only about 10% of the variation of case data can be attributed to the temperature effect and time trend.

## Conclusion

By modelling weekly temperature data and data of infectious diseases we showed that the rise in ambient temperature is associated positively with an increase in the occurrence of *S.* Enteritidis, *C. jejuni* and *C. coli* infections in German areas. These data have been not analysed for Germany so far. The temperatures (with three-week moving average) at lag times four to five weeks together with a linear trend explained more than 10% of variation in the SE cases in Berlin and Munich. Lack of knowledge of the time point of infection, incubation time, as well as reporting delays are major sources of uncertainty in this study of temporal association between ambient temperature and case incidence. Higher ambient temperatures are already a concern both on the farm and during food processing. Public health authorities should consider increases in the average ambient temperature as an early indicator for raises in numbers of foodborne infections with a four- to six-week delay. Increased surveillance during this time may improve the punctual implementation of preventive measures. Preventive measures for reducing the *Salmonella* incidence and abundance during primary production could be adequate to mitigate the increased disease risk during the warm season. Additionally, process hygiene during slaughter, processing, wholesale, and retail sale could be improved. Finally, the authors recommend intensified and improved consumer education regarding the health hazards of consuming foods potentially contaminated with *Salmonella* or *Campylobacter*, especially at the beginning of the outdoor season.

## Additional Information

**How to cite this article**: Yun, J. *et al.* Association between the ambient temperature and the occurrence of human *Salmonella* and *Campylobacter* infections. *Sci. Rep.* **6**, 28442; doi: 10.1038/srep28442 (2016).

## Supplementary Material

Supplementary Information

## Figures and Tables

**Table 1:** Percent of variance explained by negative binomial regression models with different predictors for the number of human *S.* Enteritidis (SE), *S.* Typhimurium (ST), *Campylobacter* (*C.*) *jejuni* (CJ) and *C. coli* (CC) cases reported in Berlin (B) and Munich (M) over a period from 2001 to 2004.

Case series	Linear trend over time^1^	Linear trend over time and temperature effect^2^
B, SE	0.1	14.4
B, ST	1.3	4.1
B, CJ	0.4	7.2
B, CC	1.4	7.9
M, SE	0.0	14.2
M, ST	0.1	4.1
M, CJ	0.4	9.7
M, CC	0.3	2.7

^1^Based on pseudo R-square of a model using the variable *week* as predictor.

^2^Based on pseudo R-square of a model using the variables *week* and *top* as predictors, the latter being a lag-optimised three-week moving average of temperatures recorded at given location.
