# Supplementary material for: Association between the ambient temperature and the occurrence of human Salmonella and Campylobacter infections
Source: Sci Rep. 2016 Jun 21;6:28442. doi: 10.1038/srep28442 (PMC4914963; doi:10.1038/srep28442)
Supplement: Supplementary Information [file srep28442-s1.pdf]

Supporting Information

Association between the ambient temperature and the occurrence of human *Salmonella* and *Campylobacter* infections

Josef Yun[1,2], Matthias Greiner[1,3],Christiane Höller[4], Ute Messelhäusser[4], Albert Rampp[4], Günter Klein[1]

[1]Institute of Food Quality and Food Safety, University of Veterinary Medicine of Hannover Foundation, Bischofsholer Damm 15, D-30173 Hannover, Germany

[2]Government of Lower Bavaria, Regierungsplatz 540, D- 84028 Landshut, Germany

[3] Federal Institute for Risk Assessment (BfR), Max-Dohrn-Str 8-10, D-10589 Berlin, Germany, and University of Veterinary Medicine of Hannover Foundation.

[4]Bavarian Health and Food Safety Authority, Veterinärstr. 2, D-85764 Oberschleissheim, Germany

This document contains supporting information regarding the statistical approaches for the analysis of reported cases numbers of foodborne infections due to *S*. Enteritidis (SE), *S*. Typhimurium (ST), *Campylobacter* (*C*.) *jejuni* (CJ) and *C. coli* (CC) over four years (2001-2004) in three study locations in Germany, Berlin (B), Munich (M) and rural areas in North-Swabian counties (G). We present here the trend analysis and the detrending of the time series data conducted prior to investigating cross-correlation (CCC) and non-parametric regression. Moreover, building and analysis of regression model for data summary are described.

Trend analysis

We consider weekly count numbers X_i, of human cases of foodborne infections for each pathogen (SE, ST, CJ, CC) reported for each of three study areas (B, M, G) where i=1, …, 212 relates to the time interval. Due to a small number of deviations from regular weekly intervals and for a more flexible modelling approach including the option for sample splitting we use the continuous time variable t_i, measured in weeks from 7 January 2001 (t_i=0) through 5 January 2005 (t_{212}=208.4). We modelled X_i using t_i as predictor using a log-linear (Poisson) regression (glm function in R (1)). To account for possible overdispersion, a generalised linear model with log link and negative binomial (NegBin) errors was used

(glm.nb from pscl package; Version 1.4.9 (2)). The Poisson and negative binomial regression models were compared using Vuang's statistic for non-nested model comparison ((3); implemented in the pscl package). Separate models were fitted for each combination of a pathogen and study location.

Table S.1 shows the extracted slope coefficient describing the linear trend with the associated *P* value for both count models. For all data sets, except B/ST and G/CC, the NegBin regression models provided significantly better fit compared to the Poisson models (Vuong's test, see Tab. S.1). According to the NegBin models, a small but significant negative trend was observed for location B (for agents ST, CJ, CC) and location M (agent CC) whereas a small but significant positive trend was seen for location M (agent CJ). Figures S.1 through S.16 display the time series data with overlaid trend line and detrendend data.

Table S.1. Linear slope parameter (log trend) and associated P values for Poisson regression models and alternative negative binomial regression models along with the statistics of Vuong's test for model comparison.

**Model**[1]	**Poisson (P value)**		**NegBin (P value)**		***Z* value, hypothesis, *P* value**[2]
B, SE	-0.001	(<0.001)	-0.001	(0.212)	-10.37, negbin>Poisson, <0.001
M, SE	0.001	(0.069)	0.001	(0.499)	-5.58, negbin>Poisson, <0.001
G, SE	-0.002	(<0.001)	-0.002	(0.067)	-6.86, negbin>Poisson, <0.001
SE	-0.001	(<0.001)	-0.001	(0.325)	-12.27, negbin>Poisson, <0.001
B, ST	-0.002	(<0.001)	-0.002	(<0.001)	-1.52, negbin>Poisson, 0.064
M, ST	-0.002	(0.024)	-0.002	(0.098)	-2.22, negbin>Poisson, 0.013
G, ST	0.000	(0.776)	0.000	(0.815)	-1.8, negbin>Poisson, 0.036
ST	-0.002	(<0.001)	-0.002	(<0.001)	-2.76, negbin>Poisson, 0.003
B, CJ	-0.001	(<0.001)	-0.001	(0.010)	-5.84, negbin>Poisson, <0.001
M, CJ	0.001	(<0.001)	0.001	(0.020)	-4.46, negbin>Poisson, <0.001
G, CJ	-0.003	(<0.001)	-0.003	(0.001)	-2.29, negbin>Poisson, 0.011
CJ	-0.001	(<0.001)	-0.001	(0.070)	-6.51, negbin>Poisson, <0.001
B, CC	-0.005	(<0.001)	-0.004	(<0.001)	-4.79, negbin>Poisson, <0.001
M, CC	-0.003	(0.001)	-0.003	(0.008)	-1.88, negbin>Poisson, 0.033
G, CC	0.021	(<0.001)	0.020	(<0.001)	-1.56, negbin>Poisson, 0.059
CC	-0.002	(<0.001)	-0.002	(0.012)	-5.19, negbin>Poisson, <0.001

[1] Locations (B, M) and agents (SE, ST, CJ, CC).

[2] Results of the z-statistic provided by Vuong's test.

Detrended time series data D_i were obtained by adding the respective NegBin model residuals (back-transformed to count scale using the inverse link function) to the grand mean of observed count data. Detrended time series data were used for calculate the cross-correlation coefficient (Fig. 2 in main text) and non-parametric regression for the temperature effect (Fig. 3 in main text).

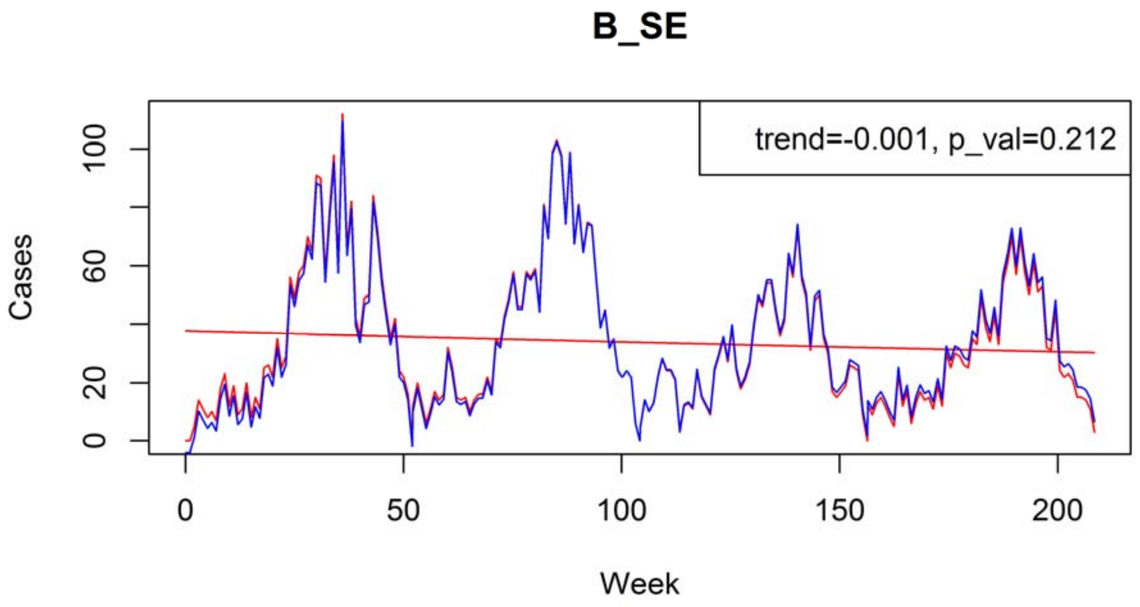

Fig. S.1. Time series for location B and pathogen SE (red graph) with overlaid linear (on the log scale) trend (red line) and overlaid detrended data (blue graph).

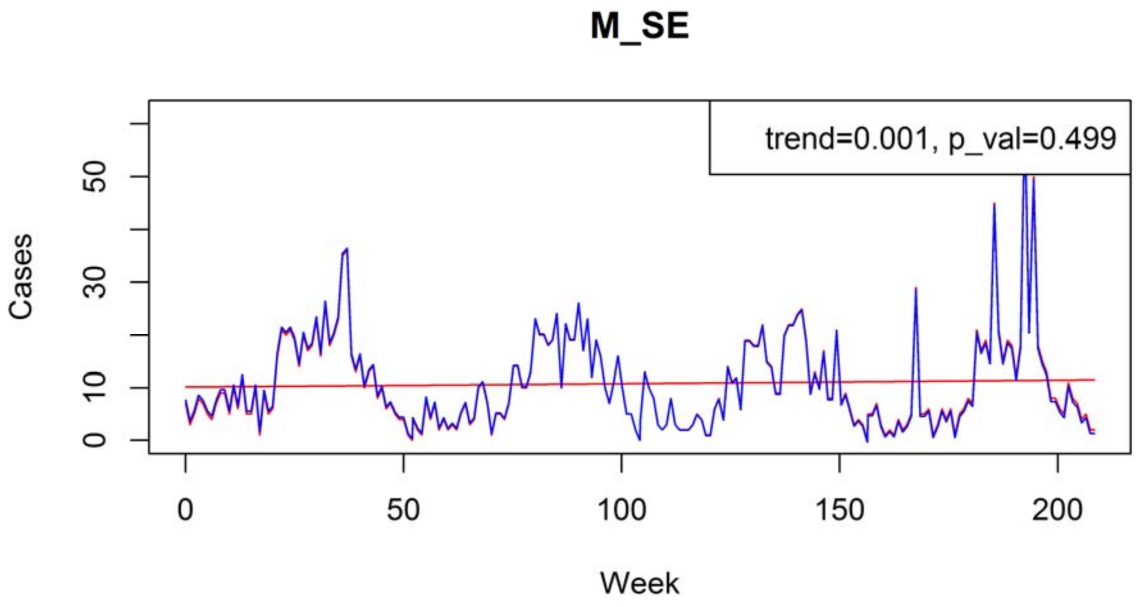

Fig. S.2. Time series for location M and pathogen SE (red graph). See Fig. S.1 for further details.

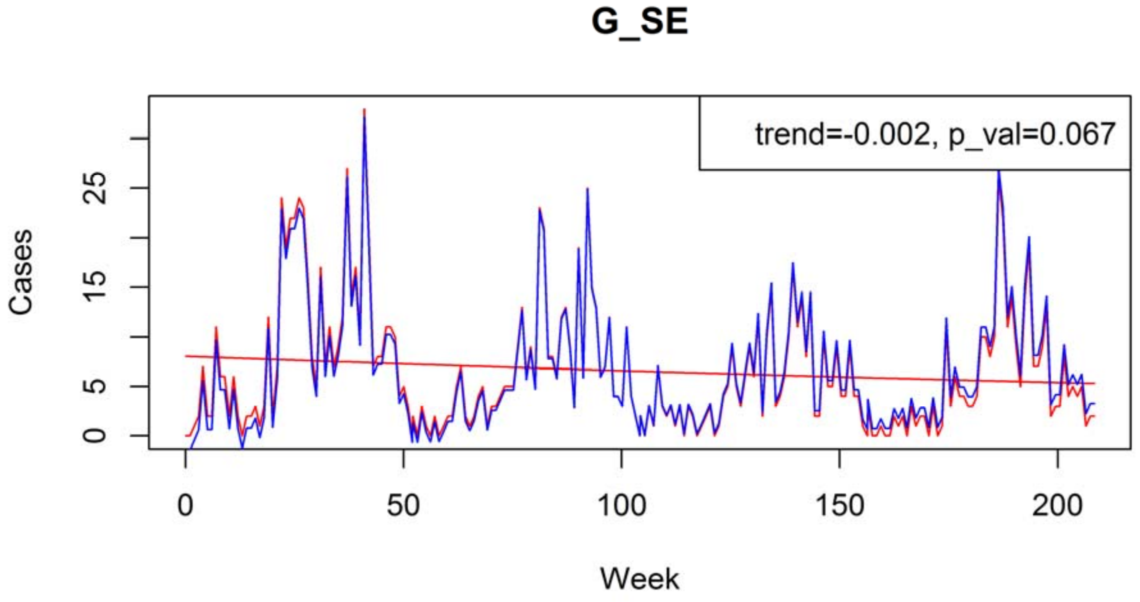

Fig. S.3. Time series for location G and pathogen SE (red graph). See Fig. S.1 for further details.

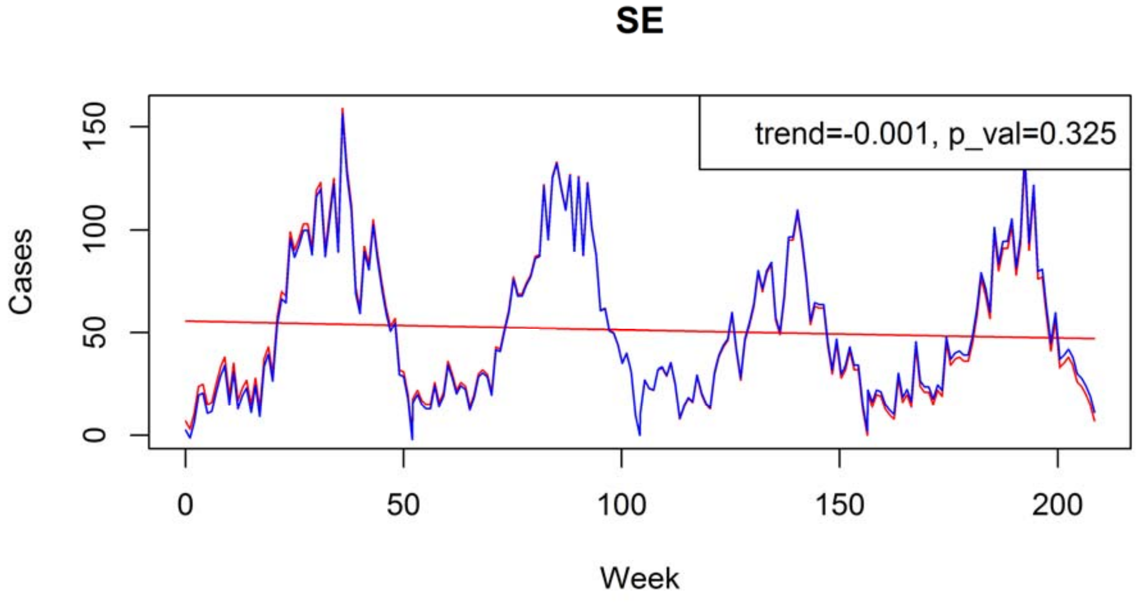

Fig. S.4. Time series for all locations combined and pathogen SE (red graph). See Fig. S.1 for further details.

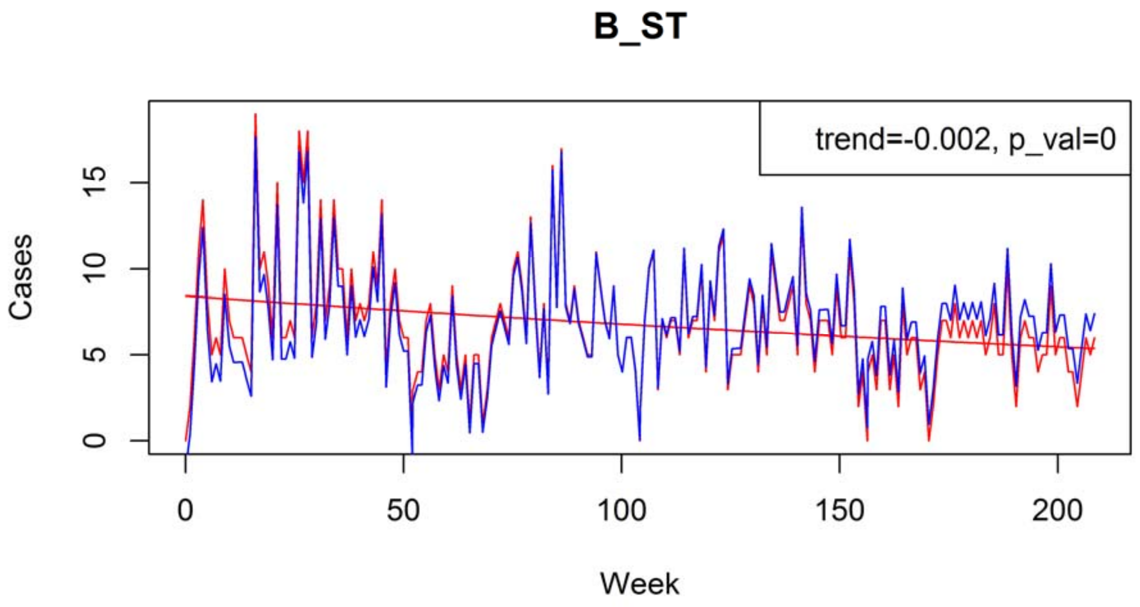

Fig. S.5. Time series for location B and pathogen ST (red graph) with overlaid linear (on the log scale) trend (red line) and overlaid detrended data (blue graph).

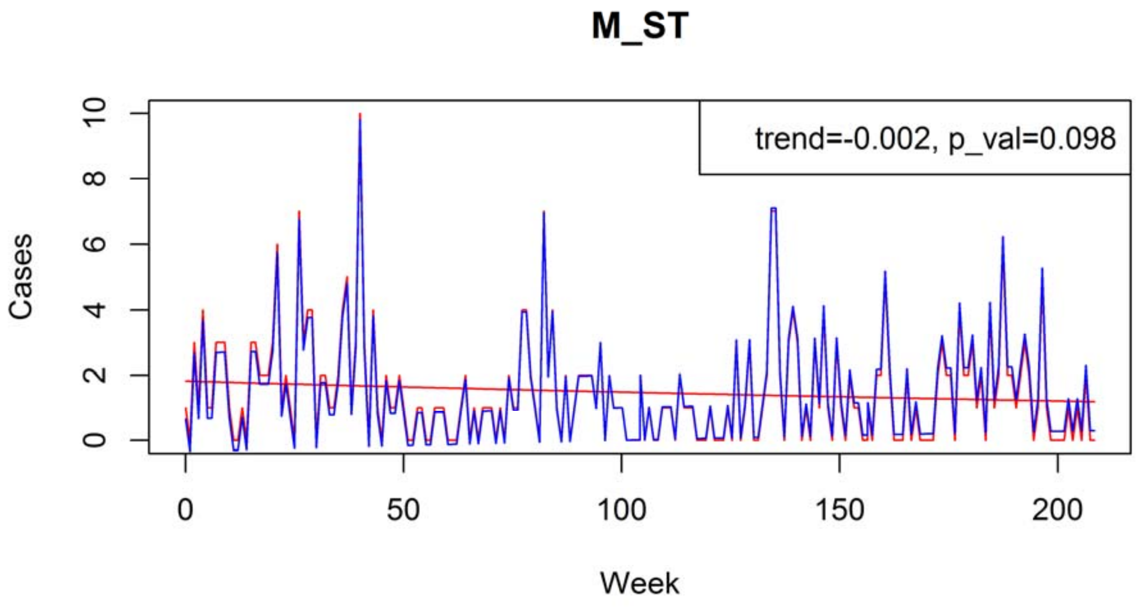

Fig. S.6. Time series for location M and pathogen ST (red graph). See Fig. S.1 for further details.

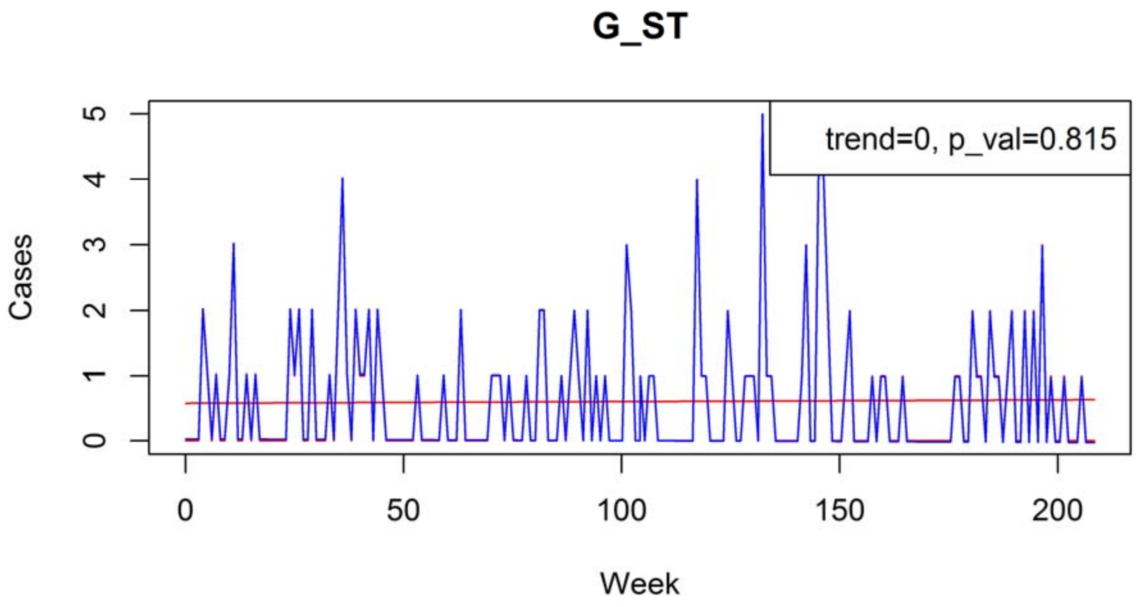

Fig. S.7. Time series for location G and pathogen ST (red graph). See Fig. S.1 for further details.

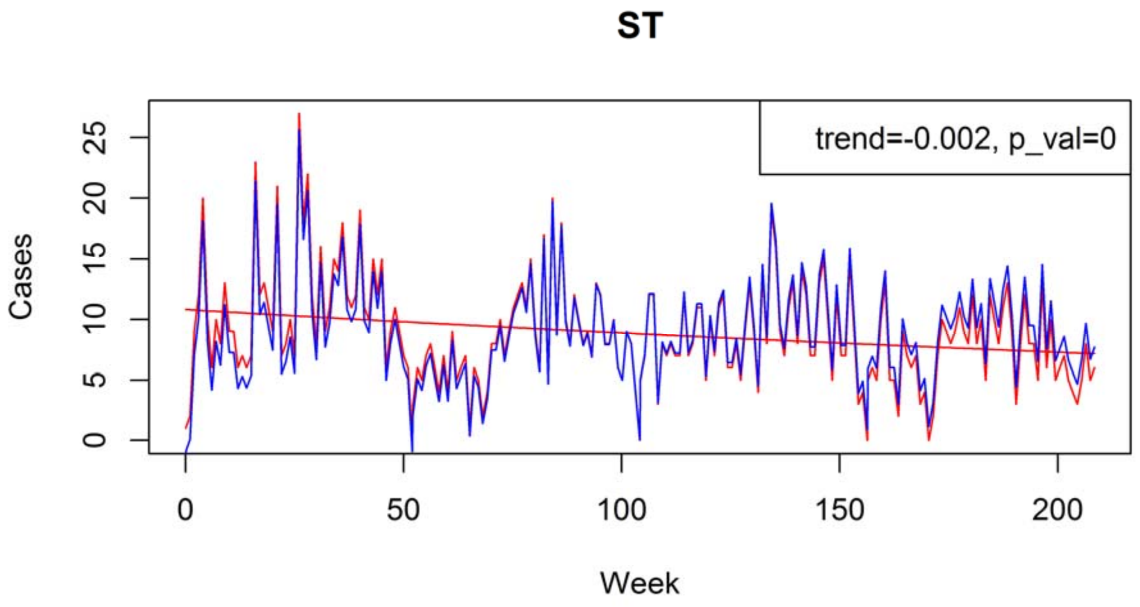

Fig. S.8. Time series for all locations combined and pathogen ST (red graph). See Fig. S.1 for further details.

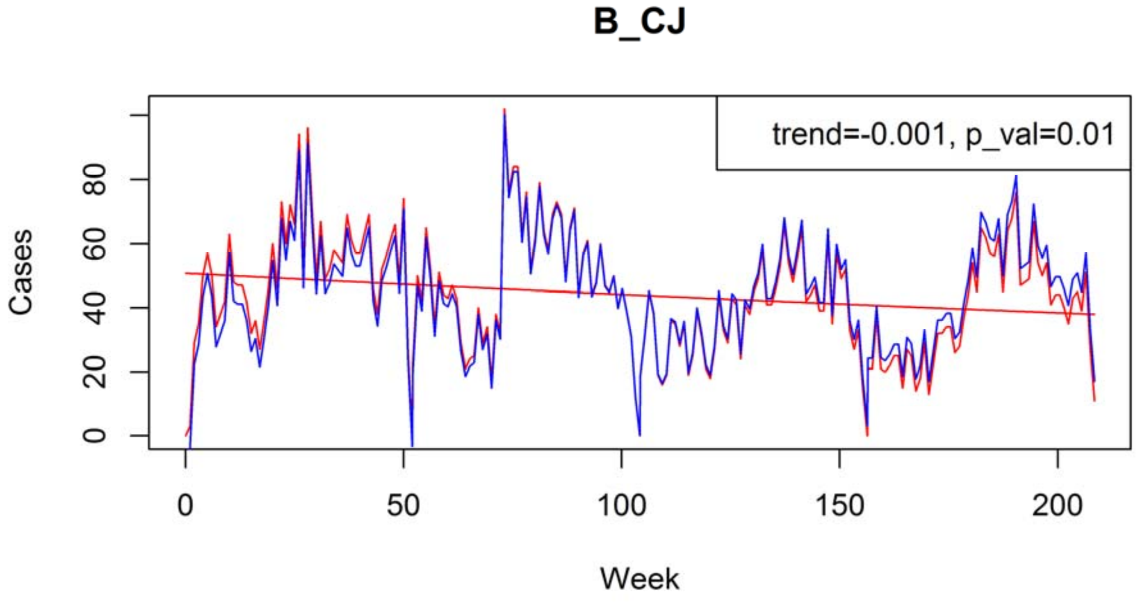

Fig. S.9. Time series for location B and pathogen CJ (red graph) with overlaid linear (on the log scale) trend (red line) and overlaid detrended data (blue graph).

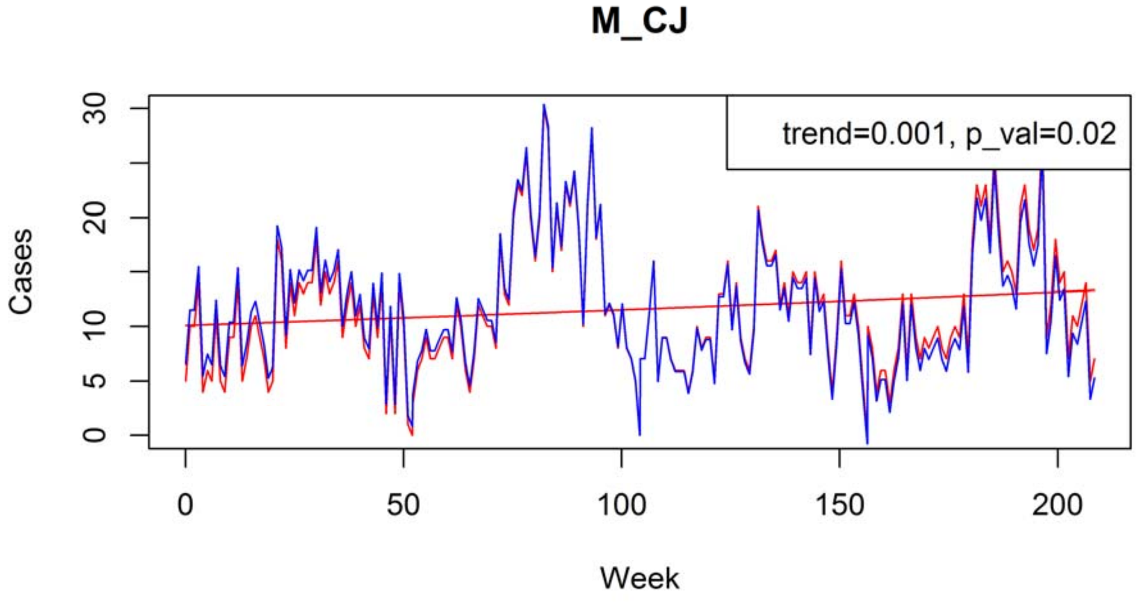

Fig. S.10. Time series for location M and pathogen CJ (red graph). See Fig. S.1 for further details.

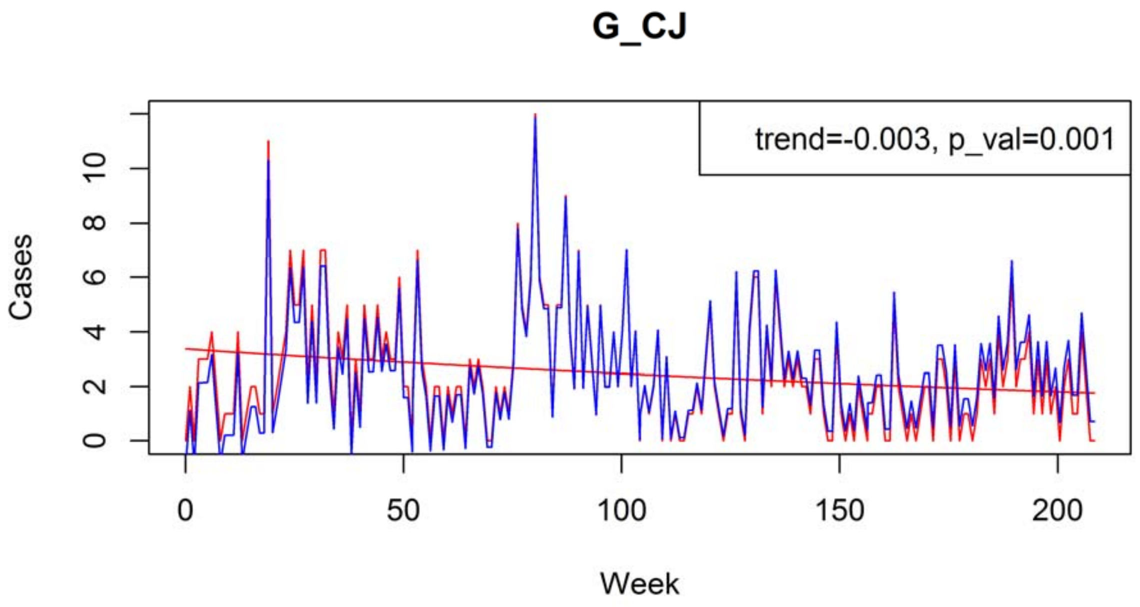

Fig. S.11. Time series for location G and pathogen CJ (red graph). See Fig. S.1 for further details.

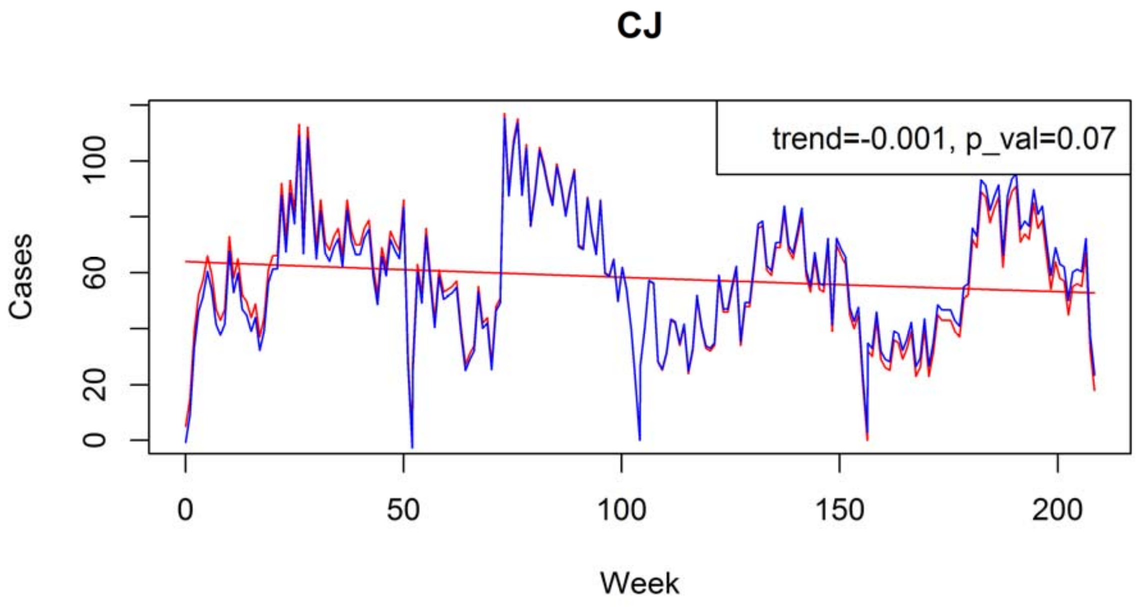

Fig. S.12. Time series for all locations combined and pathogen CJ (red graph). See Fig. S.1 for further details.

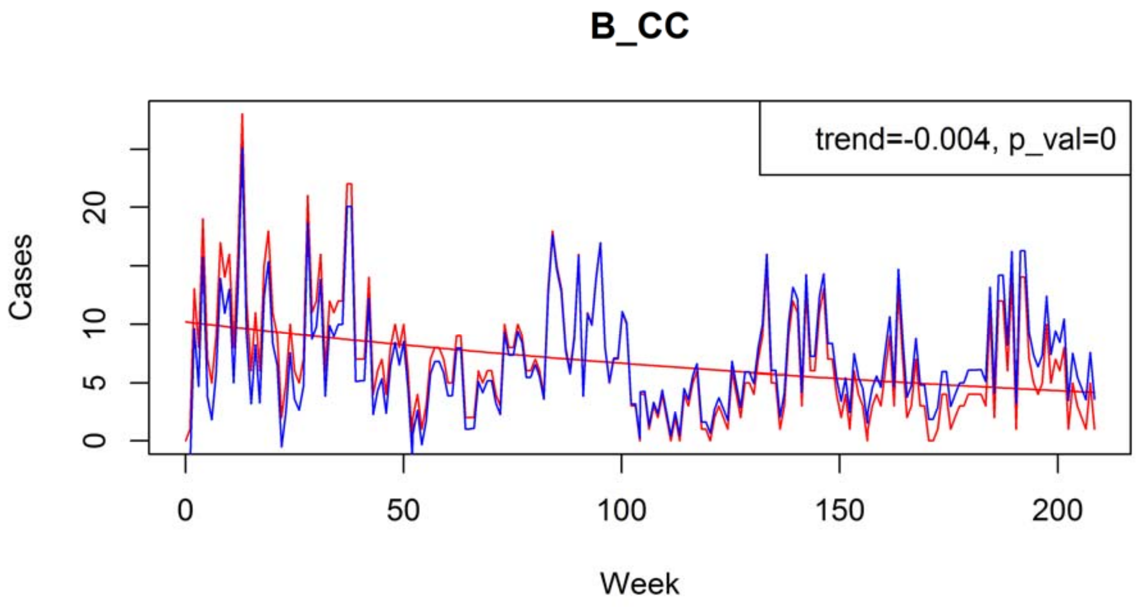

Fig. S.13. Time series for location B and pathogen CC (red graph) with overlaid linear (on the log scale) trend (red line) and overlaid detrended data (blue graph).

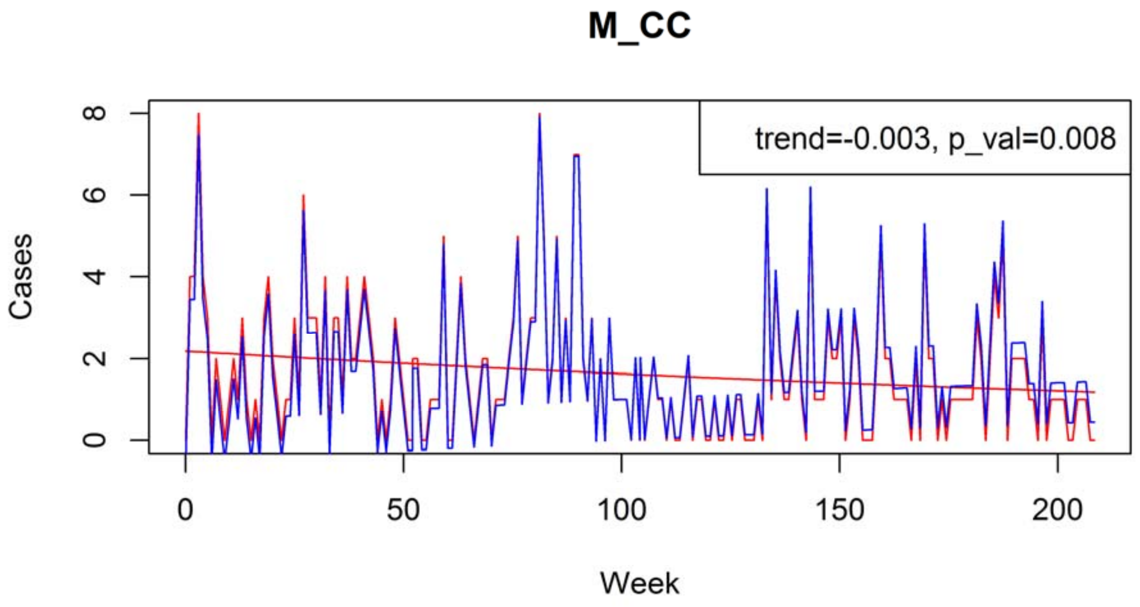

Fig. S.14. Time series for location M and pathogen CC (red graph). See Fig. S.1 for further details.

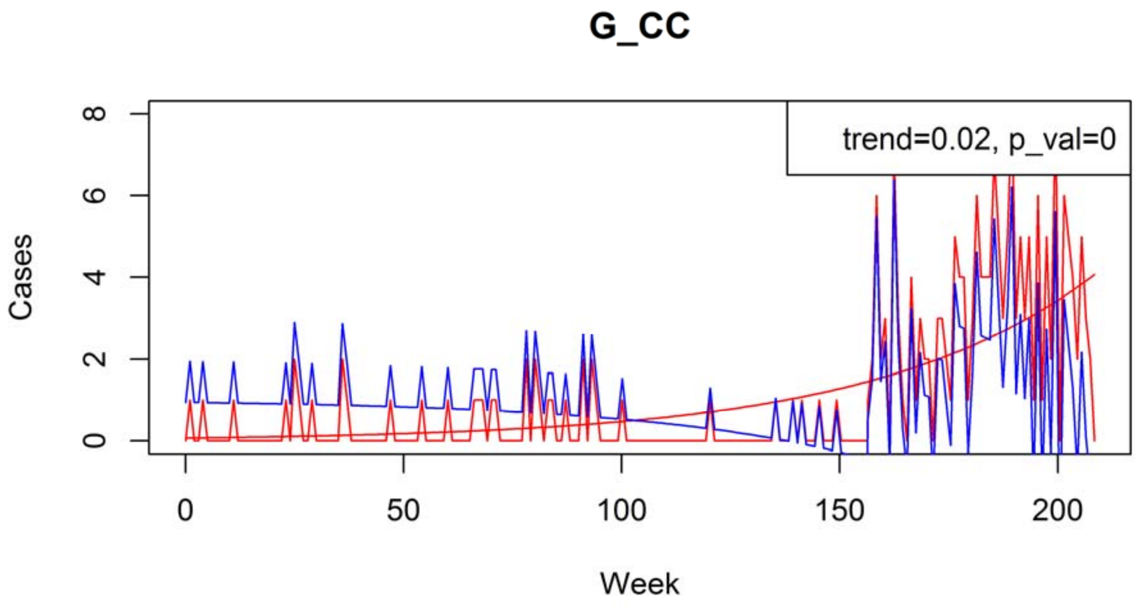

Fig. S.15. Time series for location G and pathogen CC (red graph). See Fig. S.1 for further details.

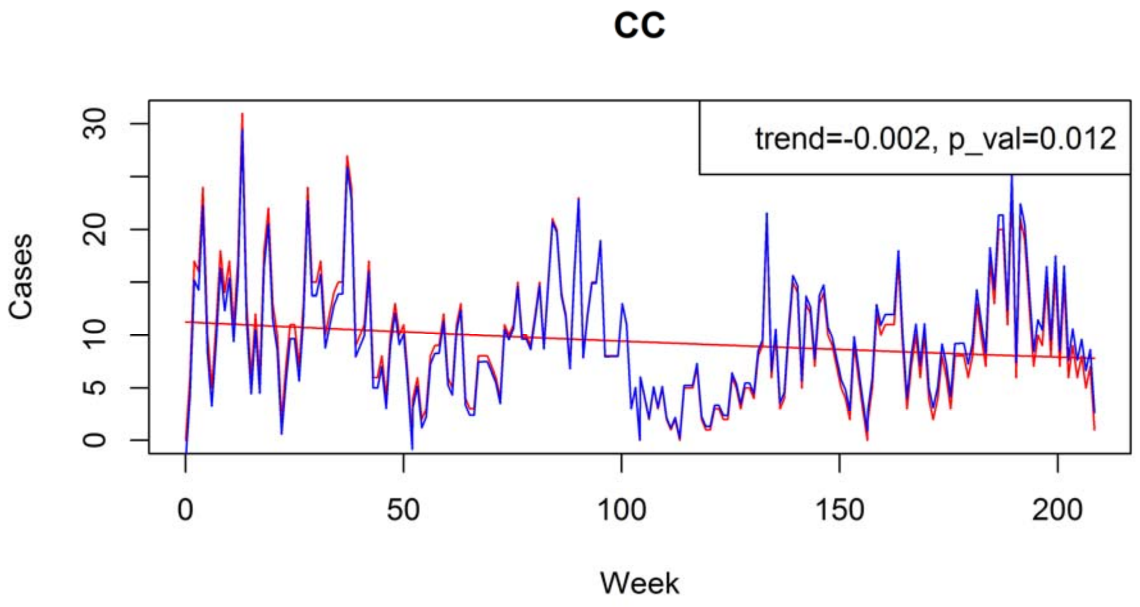

Fig. S.16. Time series for all locations combined and pathogen CC (red graph). See Fig. S.1 for further details.

Building of the final model

A data set was constructed using the observed number of cases (X_i), categorical variables for the bacterial agent (bac={SE, ST, CJ, CC}, with SE as reference) and the location (loc={B, M}, with B as reference; rural area data were excluded due to small case numbers). Eight temperature variables, *t0*, *t1*, …, *t7*, were defined as the mean temperate measured at

the respective location at lags of 0, 1, …, 7 weeks, respectively. Furthermore, the temperature variables *t1ma*, *t2ma*, *t3ma*, *t4ma*, *t5ma*, *t6ma* were constructed as the moving average (given week, one week before and one week later) of mean temperatures at the respective location at lags of 1, 2, …, 6 weeks, respectively. The data set was then split into one training set of 50 % randomly chosen observations for each combination of location and bacterial agent and one estimation set consisting of the remaining observations. The number of rows in the training set was 832 after removal of any row with missing data on any lagged temperature variable.

For model building, we fitted NegBin models to the training data for each combination of location and bacterial agent, using the week variable and in turn one out of fourteen temperature variables described above as predictors. According to Akaike's information criterion (AIC, lower values indicate better fit; Fig. S.17), the models using the moving average temperature provided a marginally better fit. For illustration, we compare models for location B and bacterial agent SE (Fig. S.17, left figure, solid line with circle). Using a mean temperature of a single week (red), the best fit is obtained with lag-5 temperature while a moving average of weekly temperatures fitted slightly better at lag-4, although this difference is not significant (Vuong's test Z=1.32, P=0.094). We chose a three-week moving average temperature as this may be a more robust predictor compared to a weekly mean.

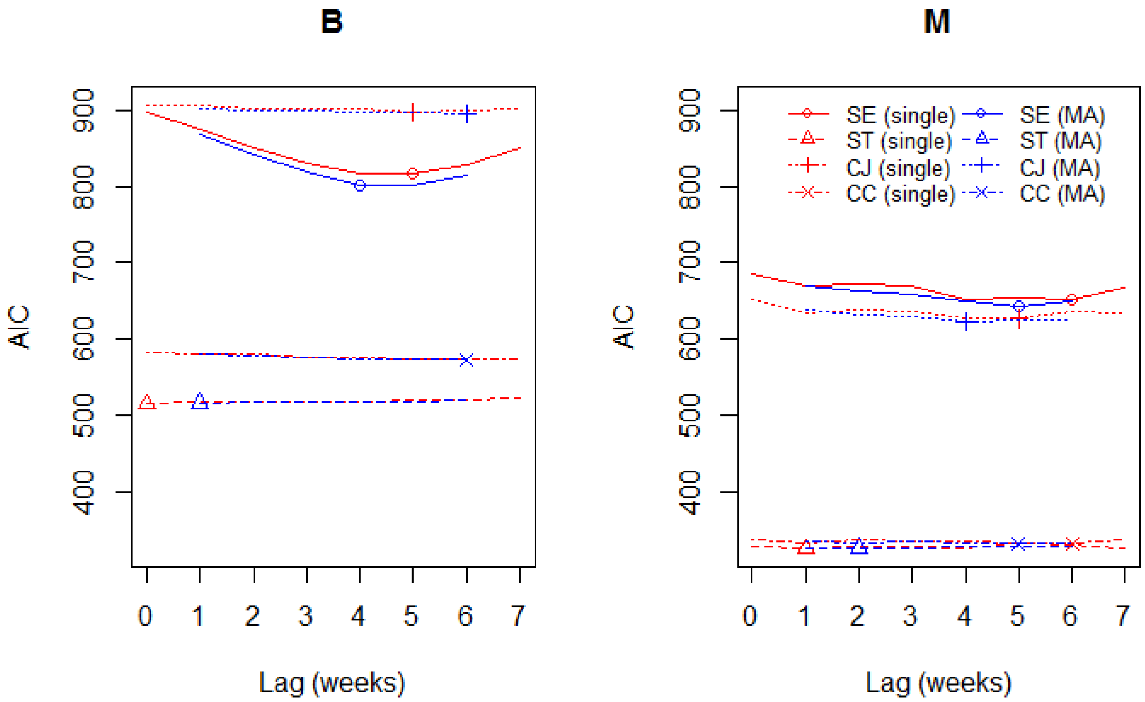

Fig. S.17. Model fit according to Akaike's information criterion (AIC) of models for analysis cases due to four bacterial agents (SE, ST, CJ, CC) in location B (left) and M (right) showing the impact of lag period (0-7 weeks) and use of single weekly mean versus three-week moving average (analysis of training data set).

Since a common optimal lag period for all combinations of location and agent could not be identified, we constructed the variable *top*, containing 3-week moving average temperature at optimal lags (B/SE: 4, M/SE: 5, B/ST: 1, M/ST: 2, B/CJ: 6, M/CJ: 4, B/CC: 6, M/CC: 5). We used this lag-optimised temperature variable in a multivariable joint model for all eight

combinations with location (*loc*={B, M}), bacterial agent (*bac*={SE, ST, CJ, CC}) and week as additional predictors and first order interaction between *bac* and all other predictors. The interaction terms allow to model the effect of main variables specifically for each bacterial species. This model has the form (in R notation)

$$\text{glm.nb(cases} \sim \text{bac*(loc+top+week),data=train).} \tag{1}$$

Model eq. (1) is considered a lag-optimised temperature model and fitted slightly but not significantly better (Vuong's test, *Z*= 1.33, *P*=0.091) compared to model eq. (2), referred here to as common-lag temperature model using *t4ma* as common temperature variable for all locations and bacterial agents

$$\text{glm.nb(cases} \sim \text{bac*(loc+t4ma+week),data=train).} \tag{2}$$

The model using *t4ma* fitted significantly better than a similar model using *t3ma* (Vuong's test, *Z*=3.377, *P*=0.0003) and better but not significantly better than a model using *t5ma* (Vuong's test, *Z*=0.78, *P*= 0.217). Therefore, the models according to eq. (1) and (2) were considered as final candidates for estimation. The final data set for estimation (*estim*) obtained by data splitting contained 808 rows after removal of any row containing missing values on either temperature variable *t4ma* or *top*, respectively.

Analysis of the final model

The lag-optimised temperature model (using the variable *top*) fitted better but not significantly better to the estimation part of the data (data=estim) than a common-lag temperature model (using the variable *t4ma*) (Vuong's test, *Z*=1.6, *P*=0.054). The parameter estimates do not deviate importantly between the two models (Tab. S.2), except one interaction term (bacCJ:locM) being borderline significant (*P*=0.047) in in the lag-optimised temperature model and non-significant (*P*=0.073) in the other model. The detailed results are provided in Table S.2, whereas Fig. 4 (main text) shows the fitted time series data. The main use of the model in our study is to derive estimates of the proportion of variance explained by the effect of time (linear trend) and the lagged temperature. The proportion of variance explained by the model was estimated using McFadden's pseudo R-square as described by Bartlett (2014). For this purpose, we considered a null model (M0) with only an intercept term, a model with only the week as predictor of linear trend (Mweek) and a model with week and the lag-optimised temperature variable (Mtop) for each combination of location and bacterial species. Using R's function logLik for extracting the log-likelihood, we obtain an estimate of the proportion of variance explained by the linear trend as

$$1 - \text{logLik(Mweek)/logLik(M0)}$$

and an estimate of proportion variance explained by the linear trend and the lag-optimised temperature as

$$1 - \text{logLik(Mtop)/logLik(M0).}$$

The results are provided in Table 1 of the main text.

Tab. S.2. Negative binomial regression models to estimate the effect of temperature at optimised lag times in comparison to a common lag-4 period for reported number of cases due to four bacterial agents (SE, ST, CJ, CC) at two locations (B, M) (analysis of estimation data set).

	Lag-optimised temperature model[1]		Common-lag temperature model[2]	
Variable	**Estimate**	**(P value)**	**Estimate**	**(P value)**
(Intercept)	2.632	(<0.001)	2.637	(<0.001)
bacST	-0.837	(<0.001)	-0.791	(<0.001)
bacCJ	0.786	(<0.001)	0.754	(<0.001)
bacCC	-0.912	(<0.001)	-0.881	(<0.001)
locM	-1.210	(<0.001)	-1.213	(<0.001)
top	0.087	(<0.001)	--	--
t4ma	--	--	0.085	(<0.001)
week	-0.001	(0.012)	-0.001	(0.020)
bacST:locM	-0.248	(0.030)	-0.253	(0.027)
bacCJ:locM	-0.157	(0.047)	-0.143	(0.073)
bacCC:locM	-0.187	(0.101)	-0.172	(0.133)
bacST:top	-0.058	(<0.001)	-0.059	(<0.001)
bacCJ:top	-0.045	(<0.001)	-0.044	(<0.001)
bacCC:top	-0.036	(<0.001)	-0.042	(<0.001)
bacST:week	-0.001	(0.411)	-0.001	(0.304)
bacCJ:week	0.000	(0.517)	0.000	(0.495)
bacCC:week	-0.003	(0.003)	-0.002	(0.006)

[1] Null deviance 6473.29 on 807 degrees of freedom, residual deviance 930.32 on 792 degrees of freedom; AIC 4439.9, Theta 11.24 (SE 1.25); 2 x log-likelihood -4405.903.
[2] Null deviance 6342.15 on 807 degrees of freedom; residual deviance 936.94 on 792 degrees of freedom; AIC 4460.2; Theta 10.79 (SE 1.20); 2 x log-likelihood -4426.227.

References

(1) R Core Team (2013). R: A language and environment for statistical computing. R Foundation for Statistical Computing, Vienna, Austria, http://www.R-project.org/
(2) Jackman S, Tahk A, Zeileis A, Maimone C and Jim Fearon. 2015. Political Science Computational Laboratory, Stanford University
(3) Vuong, Q.H. 1989. Likelihood ratio tests for model selection and non-nested hypotheses. Econometrica. 57:307-333
(4) Bartlett J (2014). R squared in logistic regression, February 8, 2014 http://thestatsgeek.com/2014/02/08/r-squared-in-logistic-regression/ (accessed January 28, 2016).
